# Evaluation of Prices for Surgical Procedures Within and Outside Hospital Networks in the US

**DOI:** 10.1001/jamanetworkopen.2022.55849

**Published:** 2023-02-13

**Authors:** Cody Lendon Mullens, Mitchell Mead, Stanley Kalata, Hari Nathan, Andrew M. Ibrahim

**Affiliations:** 1 Department of Surgery, University of Michigan, Ann Arbor; 2 Center for Healthcare Outcomes and Policy, University of Michigan, Ann Arbor; 3 Taubman College of Architecture and Urban Planning, University of Michigan, Ann Arbor

## Abstract

This economic evaluation examines variations in prices for surgical procedures under the Hospital Price Transparency Rule at hospitals within and outside hospital networks in the US.

## Introduction

Centers for Medicare & Medicaid Services (CMS) enacted the Hospital Price Transparency Rule in 2021^1^ to improve price transparency and make health care services more shoppable. This policy requires hospitals receiving federal funding to release prices for numerous health care services, including 16 surgical procedures.^1^ These prices have drawn increased attention by federal regulators who are evaluating the tradeoffs of hospital mergers and consolidations.

Whether negotiated rates differ across independent vs network hospitals remains unknown. On one hand, facilities within hospital networks may have greater market share, allowing them to negotiate higher rates with payers. On the other hand, being part of a network may allow for economies of scale, where large networks provide services at lower prices. This study was designed to evaluate variation in prices for surgical procedures under the Hospital Price Transparency Rule at hospitals within vs outside a hospital network.

## Methods

This study was exempted from review and the requirement of informed consent by the University of Michigan Institutional Review Board because it did not include human participants. Commercially negotiated prices for health care services were obtained from the Turquoise Health Database, which aggregates and processes price data published by US hospitals in accordance with the Hospital Price Transparency Rule between policy enactment on January 1, 2021, and September 27, 2022. *Current Procedural Terminology* codes were used to identify 16 surgical procedures and their negotiated prices for each payer at a given hospital. Hospital participation within a network was determined using American Hospital Association Annual Survey data. To account for potential differences in cost of living across the US, prices were adjusted using the CMS wage index.^2^ For each procedure, we determined negotiated prices at each hospital. Comparisons based on network participation were performed using Wilcoxon-Mann-Whitney tests. All data and statistical analysis were performed using Stata, version 16.0 (StataCorp LLC). The study followed the CHEERS reporting guideline.

## Results

A total of 3195 hospitals reported prices and were included in this analysis. For 15 of the 16 procedures, the median negotiated price was significantly higher at facilities within networks compared with independent hospitals (Table). Median price for shoulder arthroscopy was 1.68 times higher at facilities within networks compared with independent hospitals ($4432 [IQR, $1611-$10 593] vs $2643 [IQR, $519-$8286]; *P* < .001). For each procedure, there was significant variation in negotiated prices (Figure). The median price for prostatectomy at facilities in hospital networks and independent facilities was $9567 (IQR, $3657-$18 944) and $8601 (IQR, $4038-$17 575), respectively.

**Table.** Median Commercially Negotiated Prices for Surgical Procedures Reported in Accordance With the Hospital Price Transparency Rule

Procedure	No. of hospitals reporting ≥1 price for procedure	Price, median (IQR), $^a^		Absolute difference in price, $	Price ratio, hospital network to independent facilities	*P* value^b^
		Facilities in hospital networks	Independent facilities			
Shoulder arthroscopy with cartilage removal	1075	4432 (1611-10 593)	2643 (519-8286)	1790	1.68	<.001
Knee cartilage removal	1454	5275 (2759-8045)	3829 (1680-7063)	1446	1.38	<.001
Inguinal hernia repair	1471	4653 (2375-8893)	3683 (1444-7225)	970	1.26	<.001
Tonsil removal	1071	4954 (2478-7068)	3981 (1360-7856)	973	1.24	<.001
Biopsy						
Colonoscopy	1693	1986 (1069-3284)	1692 (844-3073)	294	1.17	<.001
EGD	1755	1678 (858-2936)	1433 (695-2511)	245	1.17	<.001
Colonoscopy, snare	1679	2008 (1073-3308)	1732 (879-2962)	275	1.16	<.001
Cholecystectomy	1519	6567 (3744-13 321)	5778 (2163-11 132)	789	1.14	<.001
Colonoscopy, diagnostic	1696	1787 (913-2914)	1562 (785-2530)	225	1.14	<.001
Prostatectomy	904	9567 (3657-18 944)	8601 (4038-17 575)	966	1.11	.09
Diagnostic heart catheterization	1264	6664 (3742-10 316)	6132 (2876-10 975)	532	1.09	<.001
EGD	1738	1572 (863-2709)	1450 (697-2512)	122	1.08	<.001
Cataract removal	1117	3466 (2124-5954)	3289 (1863-5937)	177	1.05	<.01
Laser cataract removal	911	1182 (645-2127)	1122 (749-2806)	60	1.05	<.001
Cesarean delivery	526	3460 (2213-6329)	3886 (2452-6679)	−426	0.89	<.01
Prostate biopsy	1554	1641 (703-3309)	2142 (992-3703)	−502	0.77	<.001

Abbreviations: CMS, Centers for Medicare & Medicaid Services; EGD, esophagogastroduodenoscopy.

^a^ All prices are adjusted based on CMS wage index.

^b^ Comparisons were made using Wilcoxon-Mann-Whitney tests.

**Figure.**
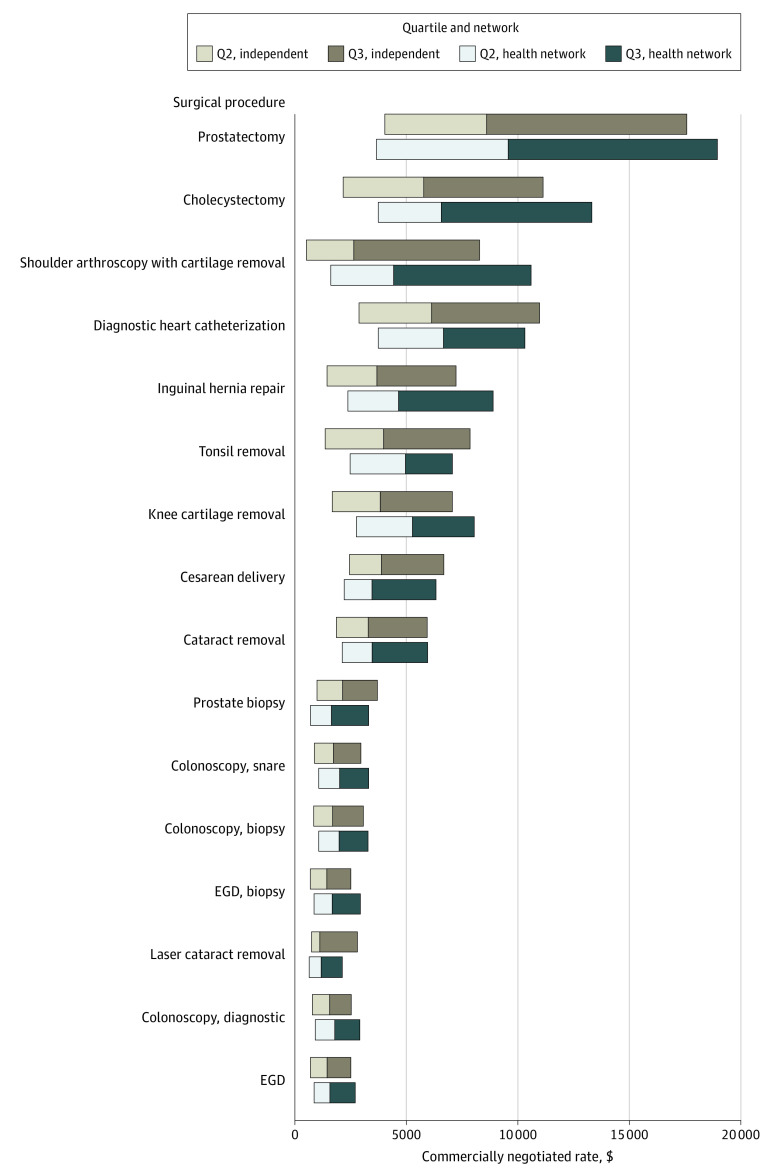
Centers for Medicare & Medicaid Services Wage Index–Adjusted Commercially Negotiated Prices for Surgical Procedures at Independent Hospitals and Those Within Networks Interquartile ranges are shown. Comparisons were made using Wilcoxon-Mann-Whitney tests. EGD indicates esophagogastroduodenoscopy; Q2, second quartile; Q3, third quartile.

## Discussion

Our economic evaluation of negotiated prices for surgical procedures at facilities participating in hospital networks vs independent hospitals had 2 principal findings. First, surgical procedures at network facilities had higher negotiated prices. Second, we noted wide variability across facilities within and outside hospital networks. Both findings build on prior work that identified commercially negotiated price variation for other health care services^3,4,5^ and extend these findings further by accounting for hospital network participation for surgical procedures.

Our findings should be interpreted in the context of important limitations. First, despite this policy being federally mandated, not all hospitals comply with it. However, compared with prior studies, our findings report more rates than previously described.^3,4,5^ Second, there may be appropriate reasons why prices of these procedures vary across hospitals that could not be identified with raw price data. We attempted to mitigate these differences by applying the CMS area wage index to account for important factors that help determine appropriate pricing for health care services. As more hospitals become compliant with this policy, it will be important to better understand the mechanisms behind these significant variations in negotiated prices for surgical care to identify areas of unwarranted variation that may be mitigated.

## Notes

Supplement.
Data Sharing Statement

## References

[1] Centers for Medicare & Medicaid Services. Hospital price transparency. Accessed October 2, 2022. https://www.cms.gov/hospital-price-transparency

[2] Centers for Medicare & Medicaid Services. Wage index. Accessed October 5, 2022. https://www.cms.gov/medicare/medicare-fee-for-service-payment/acuteinpatientpps/wageindex

[3] Haque W, Ahmadzada M, Allahrakha H, Haque E, Hsiehchen D. Transparency, accessibility, and variability of US hospital price data. JAMA Netw Open. 2021;4(5):e2110109. doi:10.1001/jamanetworkopen.2021.10109 33988709 PMC8122228

[4] Berkowitz ST, Siktberg J, Hamdan SA, Triana AJ, Patel SN. Health care price transparency in ophthalmology. JAMA Ophthalmol. 2021;139(11):1210-1216. doi:10.1001/jamaophthalmol.2021.3951 34617970 PMC8498928

[5] Jiang JX, Makary MA, Bai G. Comparison of US hospital cash prices and commercial negotiated prices for 70 services. JAMA Netw Open. 2021;4(12):e2140526. doi:10.1001/jamanetworkopen.2021.40526 34932108 PMC8693213

