# Supplementary material for: Evaluation of Prices for Surgical Procedures Within and Outside Hospital Networks in the US
Source: JAMA Netw Open. 2023 Feb 13;6(2):e2255849. doi: 10.1001/jamanetworkopen.2022.55849 (PMC9926315; doi:10.1001/jamanetworkopen.2022.55849)
Supplement: Supplement. — Data Sharing Statement [file jamanetwopen-e2255849-s001.pdf]

Data Sharing Statement

Mullens. Evaluation of Prices for Surgical Procedures Within and Outside Hospital Networks in the US. *JAMA Netw Open*. Published February 13, 2023.
doi:10.1001/jamanetworkopen.2022.55849

Data
Data available: No

Additional Information
Explanation for why data not available: Primary raw data belongs to Turquoise Health.
